# AI-driven discovery of minimal sepsis biomarkers for disease detection and progression: precision medicine across diverse populations

**DOI:** 10.3389/fmed.2025.1521827

**Published:** 2025-07-01

**Authors:** Qiyuan Su, Jingtao Huang, Yunlong Zhang, Zhou Liu, Zhihua Lv, Chunming Zhang, Chengxiu Ling, Hanwen Su, Liying Zhan, Zhengjun Zhang

**Affiliations:** ^1^Wisdom Lake Academy of Pharmacy, Xi’an Jiaotong-Liverpool University, Suzhou, China; ^2^Department of Clinical Laboratory, Institute of Translational Medicine, Renmin Hospital of Wuhan University, Wuhan, China; ^3^Department of Critical Care Medicine, Renmin Hospital of Wuhan University, Wuhan, China; ^4^Department of Statistics, University of Wisconsin, Madison, WI, United States; ^5^School of Economics and Management, and MOE Social Science Laboratory of Digital Economic Forecasts and Policy Simulation, University of Chinese Academy of Sciences, Beijing, China; ^6^AMSS Center for Forecasting Sciences, Chinese Academy of Sciences, Beijing, China

**Keywords:** gene interaction, biomarkers, AI, disease detection, progression

## Abstract

**Background:**

Sepsis biomarker research over the past 30 years has been plagued by the use of wrong animal models and inappropriate patient selections, leading to the failure of translating findings into precision medicine. Thousands of sepsis-related gene biomarkers have been published, but this excess hinders medical advancement because (1) an overwhelming number of genes make targeted drug development and precision medicine unfeasible; (2) many biomarkers lack cross-cohort validation, rendering them clinically unhelpful. Our goal is to identify a highly informative, single-digit set of sepsis biomarkers to advance precision medicine.

**Methods:**

We conducted large-scale research on heterogeneous populations, including patients with sepsis, severe sepsis, and septic shocks, and collected plasma samples from 32 sepsis patients and 18 healthy controls at Renmin Hospital of Wuhan University, China. RNA was isolated using the HYCEZMBIO Serum/Plasma RNA Kit, and RT-qPCR was performed on the Roche Light Cycler 480 platform. An AI-based max-logistic competing classifier was applied across 11 cohorts with thousands of samples, using both self-designed and public datasets to identify the most critical sepsis biomarkers.

**Results:**

Our analysis highlights CKAP4, FCAR, and RNF4 as key genetic drivers in sepsis-related variations. In whole blood, NONO is crucial for immune response, while in plasma, PLEKHO1 and BMP6 reveal further genetic heterogeneities. Pediatric patients also exhibit significant contributions from RNASE2 and OGFOD3. These genes form the most effective miniature set of biomarkers.

**Conclusion:**

Achieving 99.42% accuracy across cohorts, this miniature set outperforms larger published gene sets. These findings provide critical insights for personalized risk assessment, targeted drug development, and tailored treatments for both adult and pediatric sepsis patients.

## Introduction

Sepsis is a major global health issue characterized by life-threatening organ failures and high mortality rates. In 2017, a global study estimated 48.9 million sepsis cases and 11 million sepsis-related deaths worldwide (1). That same year, the WHO highlighted sepsis as a critical health priority, predicting its incidence to rise with the aging global population (2). Survivors often face long-term impairments and increased mortality rates post-discharge (3). Sepsis is particularly burdensome in low to middle-income countries, contributing to over 40% of all-cause mortality in some areas (1).

Sepsis results from an abnormal host response to infection, leading to multiple organ dysfunction (MODS) or multiple organ failure (MOF). Many patients suffer from long-term complications even the acute infection is treated with antibiotics (1). Unlike localized infections, sepsis causes a systemic, maladaptive response, often resulting in remote multiorgan failures (4). Factors like infection source and pathogen- or patient-specific variables influence its manifestation (5). Research indicates a typical pathophysiological pattern in sepsis, with over 80% of the transcriptomic response in leukocytes being independent of the infection source or pathogen (6). This suggests a shared transcriptomic pattern among sepsis patients, offering a potential target for personalized strategies. Although an updated international consensus on sepsis was introduced in 2017, the genomic pathology remains unclear (7). However, advances in sequencing technology and analytical platforms bring hope for future genomic studies on sepsis.

Traditional transcriptomic studies have used classic approaches like fold changes or conventional machine learning to identify differentially expressed genes (DEGs) between sepsis patients and healthy individuals (8–16). These approaches often result in large panels of DEGs, sometimes exceeding the number of samples. For instance, pioneering genomic studies at Cincinnati Children’s Hospital identified over 1,000 DEGs in pediatric sepsis patients (14, 15). Later studies on adult cohorts from Europe, America, and Australia refined the DEGs to fewer than 100 genes, though they still lack consensus on the reported DEGs (8, 13, 16). This inconsistency hinders the development of genetic treatments for sepsis, and the wrong animal models and the inappropriate selections of patients contribute to the failure of 30 years in sepsis researches (17). Thus, it is crucial to identify a concise and more accurate set of DEGs.

The evolution of quantitative medical research, fueled by advancements in computing power, brings artificial intelligence (AI) to the forefront. However, current AI models often function as black boxes, with their computational processes remaining opaque (18, 19). Additionally, AI training is often biased due to high costs limiting it to high-income settings, and the underrepresentation of pediatric data due to ethical challenges (20). A newly developed machine learning model shows promise in addressing above issues (21). This model, which has demonstrated advanced capabilities in cancer DEG recognition and subtype classification (22), aims to integrate new genomic evidence of sepsis into a concise and interpretable biological framework. Utilizing the max-logistic competing risk factors framework, this model can accurately identify a small set of critical DEGs and explain their interactions. It has proven effective in modeling various cancers and COVID-19 (22–25).

This study examined twelve datasets, including 1876 samples (1,572 sepsis and 304 control), covering diverse socioeconomic and ethnic groups, including pediatric patients highly susceptible to sepsis mortality (8–16). Among the first 11 datasets containing heterogeneous populations, including whole blood, plasma, adults, and pediatrics, three panels of five or fewer DEGs, with a common three-gene core, were identified. The first panel, consisting of four genes from adult cohorts’ whole blood gene expression data (*n* = 1,413), included CKAP4, FCAR, RNF4, and NONO, achieving near-perfect classification accuracy. The second panel, from pediatric cohorts (*n* = 287), included RNASE2 and OGFOD3 in addition to the core genes, achieving 100% accuracy. The third panel, from adult plasma samples (*n* = 106), included PLEKHO1 and BMP6 alongside the core genes, also achieving 100% accuracy. The twelfth dataset is a gene expression profiling dataset of peripheral blood mononuclear cells (PBMCs), which differs from the whole blood and plasma cohorts. Additional genes are needed to reach nearly 100% accuracy.

These gene panels demonstrated exceptional sensitivity and specificity, achieving 100% accuracy in 9 out of 11 datasets and over 95% in the remaining two. This new biological model offers a robust tool for identifying sepsis gene variations, representing the highest-performing sepsis biomarkers in the literature. The findings suggest that many previously published genes may be redundant or misleading, emphasizing the need for concise and precise biomarkers to advance precision medicine in sepsis (17, 22–26).

## Materials and methods

**Figure 1:**
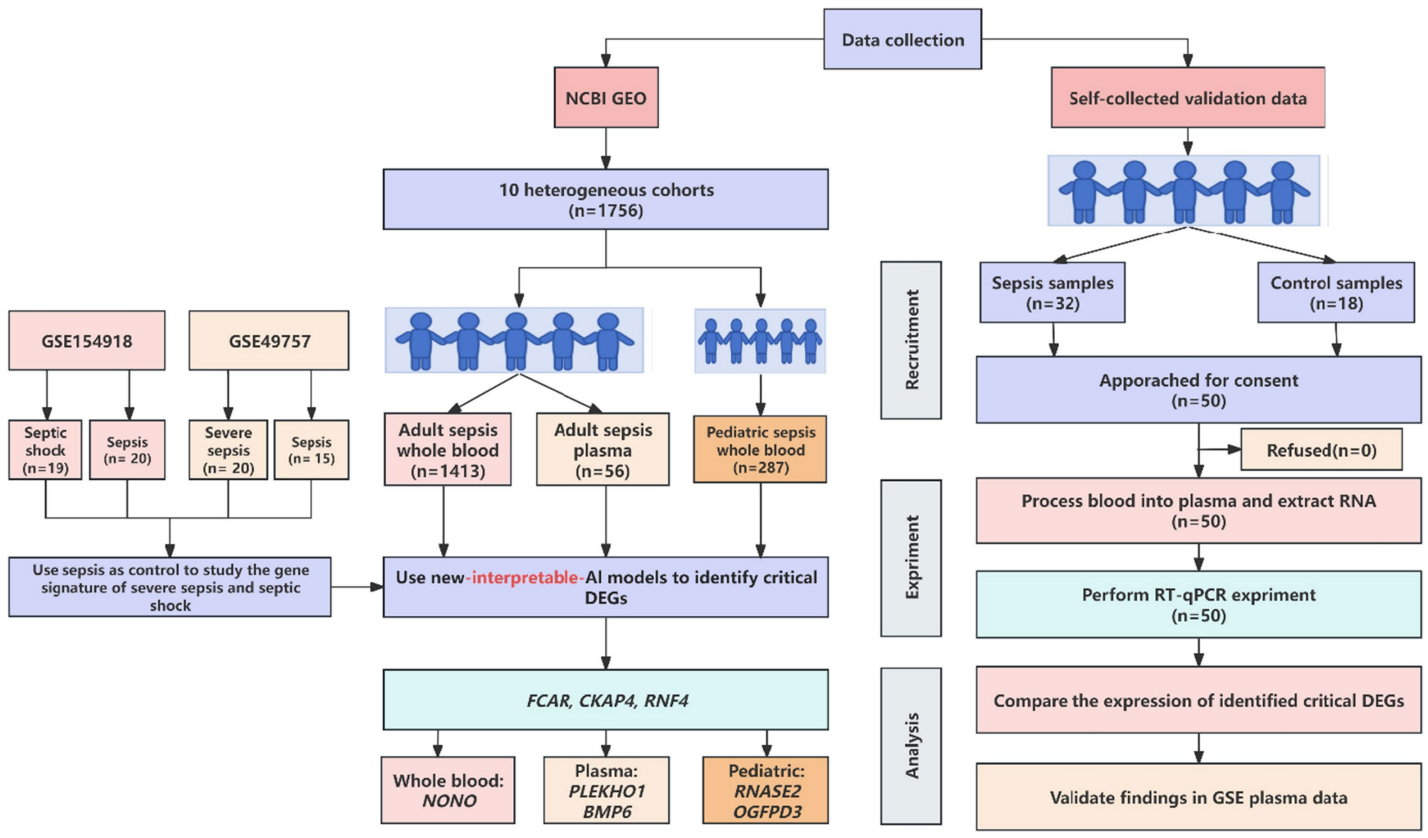
Schematic workflow of new AI-driven discovery of most effective biomarkers for sepsis patients.

Our study differs from orthodox clinical studies in the literature, which focused on rigorous experiment design while only running basic analysis on standard software. In most cases, the basic analysis was sufficient to answer the questions in completed studies in the literature. However, we argue that there is information that is overlooked, and we can extract it from public data with more advanced methods. In this section, we present both our new experimental protocol and advanced analytical method. The schematic flow of our study design is presented in Figure 1.

### Public data acquisition

This study utilized 10 public datasets from the National Center for Biotechnology Information’s (NCBI) Gene Expression Omnibus (GEO) database, using keywords “sepsis,” “septic shock,” and “homo sapiens.” Two datasets (GSE9692, GSE13904) were from pediatric cohorts in the US, while the others were adult cohorts from the US, Australia, France, Spain, and Germany. A validation dataset was self-collected from a Chinese cohort. An overview of these datasets is provided in Table 1.

**Table 1:** Basic Information of public datasets and self-collected validation set.

Datasets	Source	Sample type	Sample size	Population
1	GSE65682	Whole blood	761 sepsis samples and 41 control samples	US adult cohort
2	GSE28750	Whole blood	21 sepsis samples and 20 control samples	Australian adult cohort
3	GSE57065	Whole blood	82 sepsis samples and 25 control samples	French adult cohort
4	GSE95233	Whole blood	102 sepsis samples and 22 control samples	French adult cohort
5	GSE69528	Whole blood	83 sepsis samples and 55 control samples	US and Thai adult cohort
6	GSE131761	Whole blood	81 sepsis samples and 15 control samples	Spanish adult cohort
7	GSE154918	Whole blood	65 sepsis samples and 40 control samples	Australian and German adult cohort
			19 septic shock samples and 20 sepsis samples*	
8	GSE13904	Pediatrics	209 sepsis samples and 18 control samples	US children cohort
9	GSE9692	Pediatrics	45 sepsis samples and 15 control samples	US children cohort
10	GSE49757	Plasma	37 sepsis samples and 19 control samples	US adult cohort
			20 severe sepsis and 15 sepsis samples*	
11	Self-collected	Plasma	32 sepsis samples and 18 control samples	Chinese adult cohort
			11 septic shock and 21 sepsis samples*	
12	GSE9960	PBMC	54 sepsis samples and 16 control samples	Australian cohort
Total			1,572 sepsis samples and 304 control samples	

Repeated collection of the same patient is treated as separate samples in computation. The samples label with “*” are used in addition comparison study between different severity of sepsis.

The first seven datasets consisted of **whole blood** samples from adult patients. The first dataset, GSE65682, was from a North American adult cohort with 761 severe pneumonia/sepsis samples and 41 healthy controls. Blood was collected from ICUs, and RNA was isolated using the PaxGene Blood RNA kit (Qiagen, Netherlands) and analyzed on the Affymetrix Human Genome U219 Array platform (8). The second dataset, GSE28750 (9), the third dataset, GSE57065 (10), the fourth dataset, GSE95233 (10), the fifth dataset, GSE69528 (11), the sixth dataset, GSE131761 (12), the seventh dataset, GSE154918 (13), were from heterogeneous cohorts with different platforms and technologies. The details of data information are stated in the Supplementary material submitted together with the main file.

Two datasets (the eighth and the ninth) were from **pediatric** patients’ whole blood samples. The eighth dataset, GSE13904, was from a North American children’s cohort with 209 sepsis samples and 18 controls. RNA was isolated using the PaxGene Blood RNA kit (PreAnalytiX, United States) and analyzed on the Affymetrix Human Genome U133 Plus 2.0 Array platform (14). The ninth dataset, GSE9692, also from a North American children’s cohort, included 30 sepsis samples and 14 controls (15).

The tenth dataset, GSE49757, consisted of **plasma** samples from a North American adult cohort with 37 sepsis and 19 control samples. RNA was isolated using the RNeasy Mini kit (Qiagen, Netherlands) and analyzed on the Illumina HumanHT-12 V4.0 expression beadchip platform (16).

Among these datasets, seven from adult whole blood samples allowed much more advanced cohort-to-cohort cross-validation (different from traditional cross-validation in the literature); the two pediatric datasets could also be cross-validated. A validation cohort was collected from Renmin Hospital of Wuhan University, Wuhan, China, comprising 32 sepsis samples and 18 healthy controls to verify findings from the only public plasma dataset. RNA was isolated using the HYCEZMBIO Serum/Plasma RNA Kit (HuiYuCheng Biotechnology, China), and gene copy counting was performed using rt-QPCR on the Roche Light Cycler 480 platform.

A total of 1,806 samples (1,518 sepsis and 288 healthy controls) were collected from diverse settings and ethnicities. This comprehensive analysis addressed the challenges of data source heterogeneity and varying study objectives, which are often overlooked in the current literature. The sepsis patient cohort includes two datasets that encompass three distinct sepsis statuses: sepsis, severe sepsis, and septic shock. This enables a deeper exploration of sepsis progression by evaluating whether genes identified in sepsis versus healthy controls also carry information relevant to disease progression, specifically by comparing severe sepsis to sepsis, and septic shock to sepsis, with sepsis serving as the control in both cases.

### Validation data acquisition

Since there was only one dataset with plasma samples, we collected another cohort to validate the findings in plasma samples. The self-collected cohort was collected at Renmin Hospital of Wuhan University, which is the designated tertiary 3A (highest level) hospital of Hubei Province and provides healthcare services to patients from diverse geographic and socioeconomic backgrounds. Any patient presenting to the Department of ICU of Wuhan University Renmin Hospital with suspected severe infection or sepsis/septic shock was recognized as a prospective participant. Blood samples of prospective participants would be collected upon administration; usually, multiple tubes would be collected and sent to the Department of Clinical Laboratory for routine diagnostic testing. Patients who were positively diagnosed with sepsis via the SOFA scoring system would be approached for consent to participate in the study (7). The Department of Clinical Laboratory would release the patient samples for research proceedings when written consent was obtained. Control samples were acquired from non-sepsis volunteers.

The enrolled participants included 32 sepsis patients (21 sepsis and 11 septic shock) and 18 healthy controls. The detailed patient characteristics and gene expression values are presented in Table 2. We noted that the ration of women in the self-collected cohort may limit the generalizability of the findings. Although some prospective epidemiology studies have reported differences in sepsis incidence and mortality in different genders, there is no consensus on whether gender is a risk factor.

**Table 2:** Patient and control’s characteristics and gene expression values.

Gender	Age	Diagnosis	Outcome	Relative gene copy count					
				NONO	FCAR	CKAP4	PLEKHO1	BMP6	RNF4
Male	70	SEPSIS	Partial recovery+ discharged	19.22617679	200.8535291	711.6381286	0.858565436	3.580100284	348.4964138
Female	69	SEPSIS + liver abscess	Recovery	21.93252488	203.6573398	852.1715043	1.664397469	3.91768119	427.5650147
Male	78	SEPTIC SHOCK +MODS	Improvement	5.876674533	40.9275386	96.00249531	0.40332088	0.757858283	118.6032719
Female	42	SEPTIC SHOCK + staphylococcus	Partial recovery+ discharged	34.65520146	139.5849898	218.2745323	2.063366359	6.797479993	435.0387196
Male	76	SEPSIS + gastric track infection	Recovery	7.185058983	72.50456866	165.995463	0.664342907	1.536875181	251.6020732
Male	52	SEPSIS + gastric track infection	Recovery	26.81558831	205.0738887	544.9575334	0.299369676	5.296355642	459.8437749
Male	97	SEPTIC SHOCK + MODS	Death	132.055447	4344.576989	5693.105028	7.464263932	21.93252488	3269.830191
Female	58	SEPTIC SHOCK + urinary tract infection	Recovery	101.1252879	1991.997332	2926.579998	0.14309052	15.18947394	1494.036833
Female	58	SEPSIS + acute pancreatitis + MODS + pneumonia + ARDS	Improvement	18.25221945	222.0899039	455.0874528	0.594603558	1.918528239	207.2172074
Female	71	SEPSIS	Recovery	78.24897777	3125.77886	1601.269029	0.873572896	6.56593287	1034.702281
Female	59	SEPTIC SHOCK	Improvement	38.45235358	704.2774109	2443.951602	3.986161051	9.9176616	2142.381957
Female	57	SEPSIS	Improvement	23.02293728	926.0844826	932.5259096	1.765405993	4.438277888	438.0646531
Female	57	SEPSIS	Refused further treatment+ discharged	32.55935015	456.6674019	861.0779292	1.366040257	1.337927555	376.1071701
Female	89	SEPSIS + gastrointestinal perforation + intra-abdominal infections + pneumonia	Recovery	13.73704698	78.24897777	176.0693527	0.664342907	2.084931522	109.8963759
Male	43	SEPSIS + gallbladder perforation	Recovery	2.353813474	2528762.297	82.71058116	0.556710809	0.526680518	23.10286713
Female	70	SEPTIC SHOCK + urinary tract infection	Improvement	3.655325801	20.32241572	48.84029469	0.216134308	0.469761375	45.72781247
Male	43	SEPSIS	Improvement	23.50669813	86.52229331	639.145241	1.938579634	2.099433367	240.5178238
Female	56	SEPTIC SHOCK	Recovery	20.32241572	20.96629446	1789.077291	0.539614118	2.128740365	455.0874528
Male	78	Severe pneumonia + COVID	Refused further treatment+ discharged	107.6347412	2012.816586	4938.988862	0.986232704	34.2967508	1398.825223
Male	78	SEPSIS + acute liver failure	Improvement	44.47738303	447.2693227	1443.144453	0.346277367	11.47164198	826.0011614
Female	83	SEPTIC SHOCK	Recovery	6.844760205	102.1821935	272.4787667	0.079384436	1.735077374	142.0248924
Male	85	SEPSIS + gastric bleeding	Death	222.8609442	7281.399244	6338.826214	2.196185628	19.8353232	2702.352201
Male	74	SEPSIS + severe pneumonia	Improvement	10.92832205	182.278425	389.3705608	1.681792831	2.070529848	152.7469751
Female	60	SEPSIS + anemia	Improvement	28.05138308	922.8804737	1413.44497	0.609205132	5.314743256	602.5762495
Female	74	SEPSIS + severe pneumonia	Improvement	136.2393834	2610.300165	9184.592511	4.331900182	37.27147477	4299.639536
Female	77	Severe fever with thrombocytopenia syndrome bunyavirus	Refused further treatment+ discharged	197.4029857	10960.30253	17991.15266	1.705269784	210.8393004	30152.70894
Male	21	SEPTIC SHOCK	Improvement	38.58585049	436.5490646	999.456523	0.024433426	8.456144324	482.7056824
Female	49	SEPTIC SHOCK	Improvement	47.17661495	1488.867858	2083.798409	2.173469725	5.917550037	903.8878682
Male	74	SEPSIS + severe pneumonia	Improvement	84.15633665	1239.033947	2460.950629	1.404444876	15.24220797	1305.150082
Female	82	SEPSIS + acute pulmonary edema	Improvement	504.9511447	4672.56818	8659.091788	0.43077308	48.00124766	4138.809125
Female	73	SEPTIC SHOCK	Improvement	28.64080227	266.8712348	685.0189081	0.270743761	7.438439541	403.101684
Female	68	SEPSIS +MODS	Death	285.0359343	6295.040743	9981.21688	0.311002913	60.54768939	4011.705539
Female	52	Healthy control	N/A	0.444421341	0.755236293	1.140763716	0.323088208	0.359733395	1.105730653
Male	59	Healthy control	N/A	0.697371833	0.526680518	2.531513188	0.47963206	0.170755032	1.536875181
Male	56	Healthy control	N/A	1.01395948	0.029769937	4.377174805	0.888842681	0.24400794	1.613283518
Male	55	Healthy control	N/A	0.615572207	2.203810232	5.296355642	1.117287138	0.519429552	4.531535541
Female	58	Healthy control	N/A	1.301341855	0.765778999	6.844760205	0.803850991	0.484644908	1.500038989
Female	55	Healthy control	N/A	1.853176124	3.363585661	24.00062383	1.494849249	1.32408891	6.190259974
Female	61	Healthy control	N/A	2.703821666	5.333194708	6.498019171	1.226884977	1.771535038	2.289448321
Male	54	Healthy control	N/A	8.0556444	10.41073484	15.03236399	0.942784536	1.409320755	10.51954208
Female	58	Healthy control	N/A	50.91433496	319.5726205	290.0182746	1.542210825	2.419988178	197.4029857
Female	55	Healthy control	N/A	35.50622311	363.2955792	344.8917957	0.117034031	4.823231311	188.7064598
Female	55	Healthy control	N/A	48.33512274	223.6346614	692.1783465	0.22298213	4.141059695	120.6764206
Female	50	Healthy control	N/A	57.08342524	256	238.8564458	2.74156561	3.434261746	19.09337189
Female	50	Healthy control	N/A	26.90868529	143.509258	481.0356476	3.171136546	1.952063522	83.28587875
Male	56	Healthy control	N/A	42.37084513	116.5657387	374.8059382	0.030395467	3.305801273	109.8963759
Male	60	Healthy control	N/A	10.81528666	21.85664411	182.9112499	0.852634892	0.373712312	30.06472797
Male	59	Healthy control	N/A	114.5632091	367.0925435	1584.706553	0.033377044	7.387058486	30.2738447
Male	54	Healthy control	N/A	8.876555777	41.78751319	226.7564849	1.152686347	0.347479555	58.68825877
Female	65	Healthy control	N/A	5.098242509	2.496661098	3.237768866	4.9588308	2.070529848	68.83164099

We used real-time quantitative polymerase chain reaction (rt-qPCR) for the gene expression study. All six genes identified in the adult plasma samples (NONO, CKAP4, RNF4, FCAR, PLEKHO1, and BMP6) were examined. We performed all experimental procedures in strictly sanitized environments, from sample preparation to gene copy counting. The protocol used for the experiments is as follows: First, we processed 3 to 5 mL of whole blood samples into plasma. Next, for RNA isolation, we processed 200 μL of participants’ plasma with the HYCEZMBIO Serum/Plasma RNA Kit (HuiYuCheng Biotechnology, China). We performed the optional centrifuge cycles; otherwise, we followed the manufacturer’s recommended protocol closely during the RNA isolation process. We performed reverse transcription with the HiScript III 1st Strand cDNA Synthesis Kit (Vazyme Biotechnology, China). We used 6 μL of RNA suspended in nuclease-free water for each participant in the reverse transcription. We closely followed the manufacturer’s recommended protocol throughout the reverse transcription procedure. Finally, we performed the rt-qPCR protocol with the Taq Pro Universal SYBR qPCR Master Mix (Vazyme Biotechnology, China) on the Roche Light Cycler 480 platform (Roche, United States). We closely followed the test kits’ recommended protocol. The program we designed for the experiment included 40 cycles of denaturing at 95°C for 10 s and heating at 60°C for 30 s. The referent used for gene copy counting was β-actin; the primers corresponding to each gene and β-actin are presented in Table 3.

**Table 3:** The primers used in RT-qPCR experiment.

Gene symbol	Forward primer	Reverse primer
CKAP4	GGAGATACAGACCTCAGCCAAGT	GCGGACCTCGGTGTAGATGT
RNF4	TTAGAGCCTGTGGTGGTTGAT	GCATTCCTCCTTGGTCTTCTTC
FCAR	ACGACGCAGAACTTGATCCG	ATGGCTGTGCCAATTTTCAAC
NONO	GGCTCCTTCCTGCTAACCA	GCTGCTCTCGTTCCTTGTG
PLEKHO1	GGGACCAGCTCTACATCTCTG	TGGAGTGGGCAAGAGTAAACT
BMP6	GGTCTCCAGTGCTTCAGATTACAA	CAATGATCCAGTCCTGCCATCC
β-actin	CTGGCACCCAGCACAAT	GGCCGGACTCGTCATACT

### Analytical method

We summarize the model in this section, with detailed explanations in the Supplementary material. The AI-based analytical model employed in our study represents a generalized form of logistic linear models. The dependent variable is binary, taking the values: (1) “sepsis” or “healthy,” (2) “severe sepsis” or “sepsis,” and (3) “septic shock” or “sepsis.” The independent variables are risk factors represented by multiple genes.

The key innovation that distinguishes our model from many traditional ones is that the risk factors are not individual genes but linear combinations of several genes. This enhancement allows the model to capture gene interactions, with the signs (+/−) of these combinations providing insight into the regulatory relationships between genes. Additionally, we developed a framework, outlined by seven rules (found in the Supplementary material), to define critical differentially expressed genes (DEGs) that guide our model’s analysis. The model evaluates every possible linear combination of genes, ultimately identifying the combination of critical DEGs with the highest risk association to the “sepsis” outcome.

Given that our study spans 11 cohorts, including patients with sepsis, severe sepsis, septic shock, and healthy controls, several pertinent questions arise: (1) How to select control group? (2) How was the analytic process that identified the transcriptional profiles established? (3) Were the cohorts pooled? (4) Were the cohorts compared? (5) How was the initial model developed?

For question (1), controls in our analysis consist of either healthy individuals or sepsis patients, with the latter serving as controls when comparing severe sepsis and septic shock cases. In published studies, the primary focus is often distinguishing sepsis patients from healthy controls, with high diagnostic accuracy reported for specific genes. However, differentiating between healthy individuals and severe sepsis patients is not particularly challenging; a clinician with moderate expertise can easily recognize the difference. What is truly needed is the ability to identify distinct subsets within the sepsis population that may respond differently to targeted therapy. Although these observations hold generally true, our selection of healthy controls differs significantly from prior research. First, as discussed in the Introduction, thousands of published genes from prior studies often fail to replicate across cohorts. Second, our identified gene set is the smallest, most informative group of biomarkers for sepsis. Third, their use in diagnosing sepsis is only a fraction of their potential utility. Fourth, this minimal gene set completely (100%) captures the genetic variability between sepsis patients and healthy controls. Fifth, it reflects genetic differences between sepsis, severe sepsis, and septic shock populations. Sixth, this set may respond uniquely to specific treatments, whereas larger gene sets may be too broad to offer focused therapeutic guidance.

For question (2), the analytical process is described in detail through the solution of the objective function (s5) outlined in the Supplementary material, followed by the corresponding procedures.

For question (3), the 11 cohorts were not pooled. Since the samples were derived from diverse ethnic groups, age categories, and experimental platforms, pooling would introduce batch effects, which could compromise the integrity of the inferences despite attempts at correction. Our max-logistic classifiers address batch effects by treating the cohorts independently in a cohort-to-cohort cross-validation framework, a method far more advanced than traditional cross-validation approaches in the literature. Relevant details are provided in the Supplementary material and our prior publications (22).

For question (4), the cohorts were not directly compared. However, we applied the most rigorous critical gene rules available in the literature (22).

For question (5), the computational procedure for model development is thoroughly documented in the Supplementary material and our previous publications (22).

## Results

### Identification of critical DEGs

After analyzing 11 datasets, we identified three panels with eight critical DEGs, all sharing a core set of CKAP4, FCAR, and RNF4. Panel one, which adds NONO, showed nearly perfect classification in seven datasets from sepsis patients’ whole blood samples, achieving 100% accuracy, sensitivity, and specificity in five and over 95% in the other two. Notably, FCAR alone had perfect classification in three datasets. Panel two includes RNASE2 and OGFOD3 and performed flawlessly in two pediatric cohorts. Panel three adds PLEKHO1 and BMP6, showing perfect classification in two sepsis plasma datasets. While literature links NONO, FCAR, BMP6, RNF4, and RNASE2 to sepsis or Systemic Inflammatory Response Syndrome (SIRS), their interactions and the roles of PLEKHO1, OGFOD3, and CKAP4 in sepsis are less documented. Findings from our self-collected dataset reinforced these results.

### Identification of classifiers based on DEGs: (1) sepsis versus healthy

**Figure 2:**
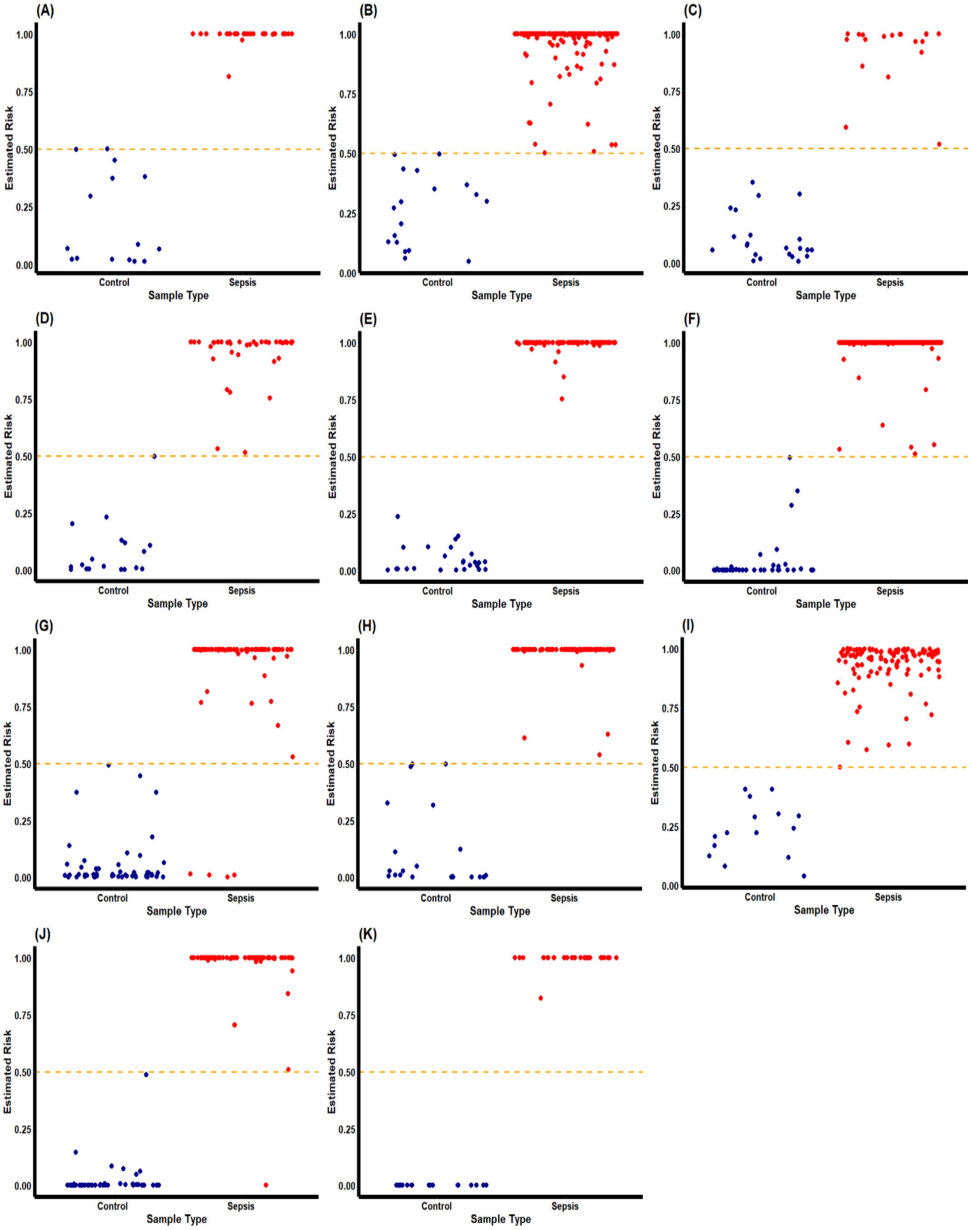
Estimated risk probability based on the final classifiers for sepsis patients and control subjects in different cohorts. These plots describe the risk probability for each subject in a cohort based on their maximum competing risk factor. Sepsis patients and control subjects are represented by red and blue dots, respectively. The orange dash-line represent the *p* = 0.5 probability threshold for our classifiers to separate “septic” and “healthy” classes. We can observe that the final classifiers has excellent accuracy in most of the cohorts. GSE9692 is labeled with **(A)**; GSE13904 is labeled with **(B)**; GSE28750 is labeled with **(C)**; GSE49757 is labeled with **(D)**; GSE57065 is labeled with **(E)**; GSE65682 is label with **(F)**; GSE69528 is labeled with **(G)**; GSE95233 is labeled with **(H)**; GSE131761 is labeled with **(I)**; GSE154918 is labeled with **(J)**; and the self-collected cohort is labeled with **(K)**.

**Figure 3:**
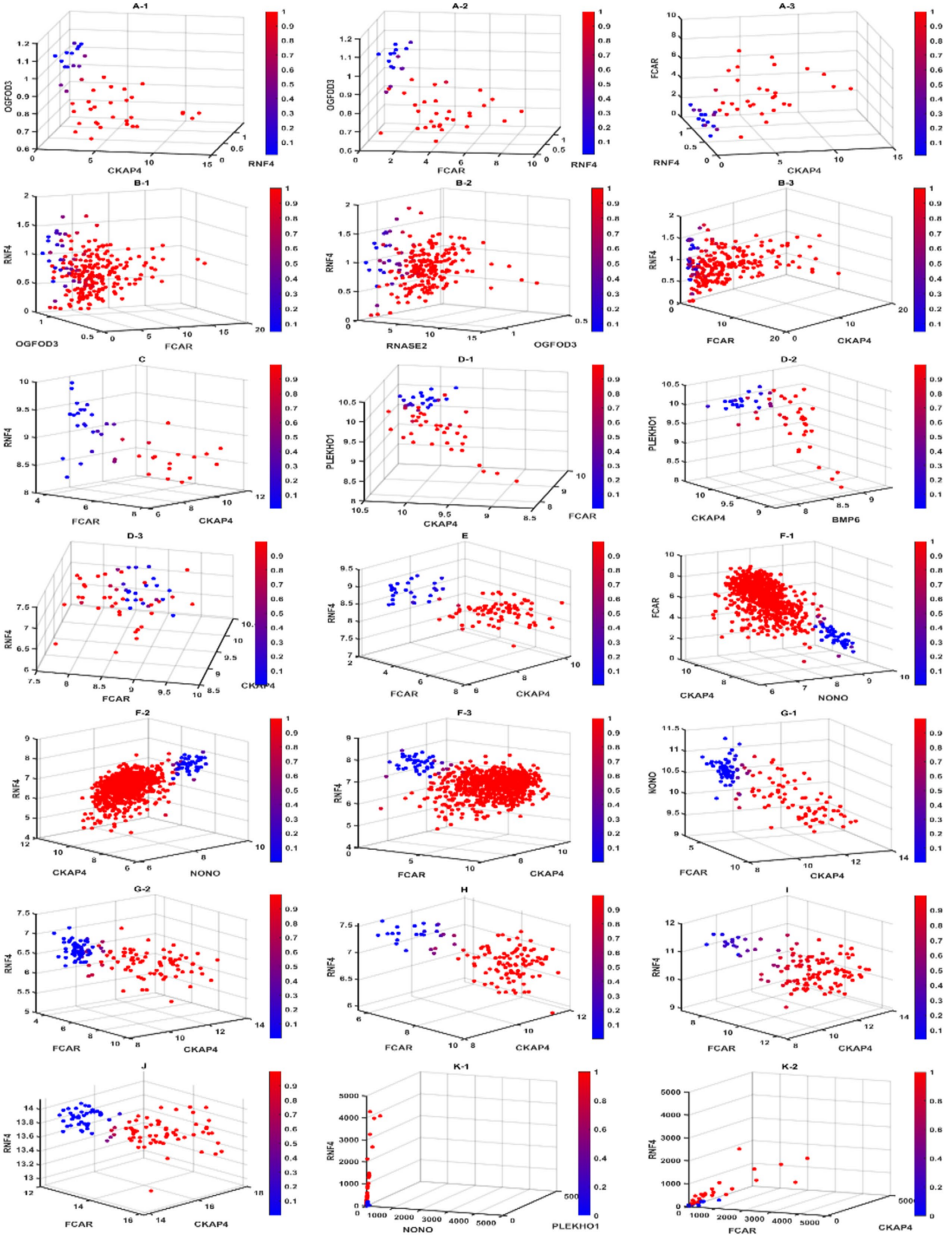
Diagnostic view of different competing risk factors. In this figure, we present the gene–gene interactions and signatures for different competing risk factors. Scatter plots labeled with the same letter contain patients from the same cohort. Subjects with a higher risk of a sepsis classification are in red, while subjects with a lower risk are in blue. We can see clear separation in all cohorts, indicating the high accuracy of our classifier model. For cohorts that required less than 3 genes in the competing risk factor classifiers, we used the 3-gene core to present the diagnostic view. GSE9692 is labeled with **(A)**. **(A-1)** to **(A-3)** are Competing risk factor 1 (CF1), Competing risk factor 2 (CF2), and 3-gene-core (core), respectively. GSE13904 is labeled with **(B)**. **(B-1)** to **(B-3)** are CF1, CF2, and core, respectively. GSE49757 is labeled with **(D)**. **(D-1)** to **(D-3)** are CF1, CF2, and core, respectively. GSE65682 is labeled with **(F)**. **(F-1)** to **(F-3)** are CF1, CF2, and core, respectively. GSE69528 is labeled with **(G)**. **(G-1, G-2)** are CF1 and core, respectively. GSE154928 is labeled with **(J)**. The self-collected cohort is labeled with **(K)**. **(K-1, K-2)** are CF1 and core, respectively. GSE28750, GSE 57065, GSE95233, and GSE131761 only required 1 gene in their competing risk classifiers are labeled with **(C,E,H,I)** respectively.

The new AI-type models (max-logistic competing risk factors) were trained to identify classifiers that discriminate sepsis samples from healthy controls (Figure 2). In the max-logistic competing risk factor model, each competing factor ( ${\mathrm{CF}}_{i},i=1,2,3$ ) is a linear combination of DEGs. The final classifiers that discriminate a sepsis sample from a control sample are presented in Table 4. The risk probability can be calculated by applying the logistic function, as shown in Equation 1. Each ${\mathrm{CF}}_{i}$ represents a gene-sepsis association and reflects how genes interact. Individual samples may have multiple ${\mathrm{CF}}_{i}\;s$ , representing the competing risk factors for that patient. The final model reports the maximum of all ${\mathrm{CF}}_{i}$ (CF_max_ = max(CF*_i_*)) for each sample. The risk probability estimated by the final model’s classifier of both sepsis and control groups is visualized in Figure 3. The model classifies samples with above 50% risk as “sepsis” (or “severe sepsis,” or “septic shock”), while those less than 50% as “healthy” (or “sepsis”), we can evaluate the model’s performance by comparing the model output with the sample’s original classification.


(1) $$\text{Risk}=\frac{e^{\mathrm{CF}._{\max}}}{1+e^{\mathrm{CF}._{\max}}}.$$

**Table 4:** The eight critical DEGs and the classifiers identified in 11 datasets.

	Data	Sepsis	Healthy	Region	Type	Classifier	Intercept	CKAP4	RNF4	FCAR	NONO	RNASE2	OGFOD3	PLEKHO1	BMP6	Accuracy	Sensitivity	Specificity
1	GSE65682	761	41	Malta	Whole blood	CF1	−2.7350	2.3794		−8.07	0.0442					5.36%	0.13%	100.00%
						CF2	8.8323	11.1401	−8.24		−4.2700					99.88%	99.87%	100.00%
						Max										100.00%	100.00%	100.00%
2	GSE28750	21	20	Australia	Whole blood	CF1	−17.7260			3.507						100.00%	100.00%	100.00%
3	GSE57065	82	25	France	Whole blood	CF1	−23.9523		5.6255							100.00%	100.00%	100.00%
4	GSE95233	102	22	France	Whole blood	CF1	−16.8988	0.6058	−4.828	6.4325						100.00%	100.00%	100.00%
5	GSE69528	83	55	United States/Thai	Whole blood	CF1	−11.4506	6.3675		1.8438	−5.6657					97.10%	95.18%	100.00%
6	GSE131761	81	15	Spain	Whole blood	CF1	−17.1026			2.0143						100.00%	100.00%	100.00%
7	GSE154918	65	40	Australia/Germany	Whole blood	CF1	30.0238	8.0139	−14.7455	3.9406						95.24%	94.94%	96.15%
7*	GSE154918	Septic shock	Sepsis	Australia/Germany	Whole blood	CF1	−25.8088	9.3795	−11.7651		2.0071					61.54%	21.05%	100.00%
						CF2	21.8625	−6.1460	−2.5326	7.3024						82.05%	63.16%	100.00%
		19	20			CF3	197.1369		−52.9581	2.5032	33.043					76.92%	63.16%	100.00%
						Max										92.31%	94.74%	90.00%
8	GSE9692	45	15	United States	Whole blood	CF1	1.7772	7.2055					−16.4062			100.00%	100.00%	100.00%
						CF1	22.6871		1.9885				−28.0567			100.00%	100.00%	100.00%
						CF1	−3.2680	5.7759	−8.34							100.00%	100.00%	100.00%
9	GSE13904	209	18	United States	Whole blood	CF1	8.3383		0.5639	0.9597			−11.1228			91.63%	90.75%	100.00%
						CF2	4.5817		−10.46			8.5348	−9.2377			90.91%	89.95%	100.00%
						Max										100.00%	100.00%	100.00%
10	GSE49757	37	19	United States	Plasma	CF1	64.0608	6.8444		−10.22				−4.7724		66.07%	48.65%	100.00%
						CF2	−5.6928	−12.743						0.8999	14.5114	91.07%	86.49%	100.00%
						Max										100.00%	100.00%	100.00%
10*	GSE49757	Severe sepsis	Sepsis	United States	Plasma	CF1	105.1717	−27.0928			14.1109			−56.3939	24.3984	97.14%	100.00%	93.33%
		20	15															
11	New Data	32	18	China	Plasma	CF1	−2.7514		1.0879		−6.5770			−9.6589		100.00%	100.00%	100.00%
11*	New Data	Septic shock	Sepsis	China	Plasma	CF1	5.4025	−0.1555		−7.7554				5.3968		81.25%	45.45%	100.00%
						CF2	−11.4285			7.0408	0.0029			−1.5894		71.88%	18.18%	100.00%
		11	21			CF3	4.1299			−9.5293				1.6554	−0.0370	75.00%	27.27%	100.00%
						Max										93.75%	81.82%	100.00%
Total	1,912	1,568	344													99.42%	99.49%	99.13%

The final classifiers are combined classifiers of individual competing factors. The gray-shaded genes and numbers are for pediatric septic cases. The green-colored genes and numbers are for plasma samples. Additionally, we compared patients with different sepsis severity in cohort GSE154918 and GSE49757, and we used sepsis as control to assess the gene signature of septic shock and severe sepsis in these patients. The results for the comparison are labeled with “*”.

Using CF_2_ in the 1st (GSE65682) dataset in Table 4 as an example, we have

${\mathrm{CF}}_{2}=8.8323+11.1401^{*}\text{CKAP}4–8.24^{*}\mathrm{RNF}4–4.2700^{*}\text{NONO}$

and ${\mathrm{CF}}_{\max}=\max\;({\mathrm{CF}}_{1},{\mathrm{CF}}_{2})$ .

In the 2nd (GSE28750), 3rd (GSE57065), 4th (GSE95233), 5th (GSE69528), 6th (GSE131761) dataset, and the 11th (self-collected) dataset, a single classifier had sufficient high power (close to or reaching 100% accuracy, 100% sensitivity, and 100% specificity) to discriminate sepsis samples from healthy controls. We note that for the 2nd, 3rd, and 6th datasets, FCAR alone was enough to act as a classifier to discriminate sepsis samples and healthy controls. Two classifiers were needed to achieve similar high power for the 8th (GSE13904), 10th (GSE49757), and 1st (GSE65682) due to insufficient sensitivity of CF_1_ in those datasets. We note that for the 1st dataset, CF_1_ was created to adjust for a unique patient, and the result was that CF_1_ for the 1st dataset had poor overall sensitivity. We are unsure if this patient had a distinctly different genetic profile from other patients in the same cohort or if there was a recording error. The 8th (GSE9692) dataset showed three classifiers each to reach high classification power with 100% accuracy. All but two datasets (5th GSE69528 and 7th GSE154918) had a maximum classifier (CF_max_) with 100% accuracy, 100% sensitivity, and 100% specificity.

### Identification of classifiers based on DEGs: (2) severe sepsis versus sepsis

As discussed in the Introduction, numerous sepsis-related genes have been reported in the literature but often lack cohort-to-cohort cross-validation. Our study successfully cross-validated a miniature gene set across 11 cohorts using healthy populations as controls, demonstrating their high informativeness and reliability as sepsis biomarkers.

To test whether this gene set remains informative in the progression from sepsis to severe sepsis, we analyzed plasma data from GSE49757, comprising 20 severe sepsis samples and 15 sepsis samples. We found that a combination of four genes (NONO, CKAP4, PLEKHO1, and BMP6) achieved a differentiation accuracy of 97.14%, with a sensitivity of 100% and a specificity of 93.33%. This indicates that the miniature gene set retains its intrinsic value, regardless of the control used. These results further confirm that this gene set is applicable for studying sepsis progression.

### Identification of classifiers based on DEGs: (3) septic shock versus sepsis

We used 19 septic shock samples and 20 sepsis samples from whole blood data in GSE154918 to test the miniature gene set. The combination of four genes (NONO, CKAP4, RNF4, and FCAR) achieved a differentiation accuracy of 92.31%, with a sensitivity of 94.74% and a specificity of 90.00%. Using our new plasma data (11 septic shock samples and 21 sepsis samples), the combinations of five genes (NONO, CKAP4, FCAR, PLEKHO1, BMP6) reached an overall accuracy of 93.75%, with a sensitivity of 81.82% and a specificity of 100.00%. Once again, these results demonstrate that the miniature gene set retains its intrinsic value, regardless of the control used. These findings further confirm the utility of this gene set for studying the progression of sepsis.

### Interpretation of gene variations and the clinical syndrome of sepsis reflected by the classifiers

In the formulas for classifiers, we can observe + and – coefficient signs for different genes. For genes in a classifier, + indicates that upregulation of that gene increases the risk of that patient being classified as “sepsis,” while − indicates that downregulation of that gene increases the risk of that patient being classified as “sepsis.” We note that many published works did not discuss the fitted coefficient signs, so their corresponding genes’ actual functions remain unclear.

#### The whole blood samples of adult patients

**Figure 4:**
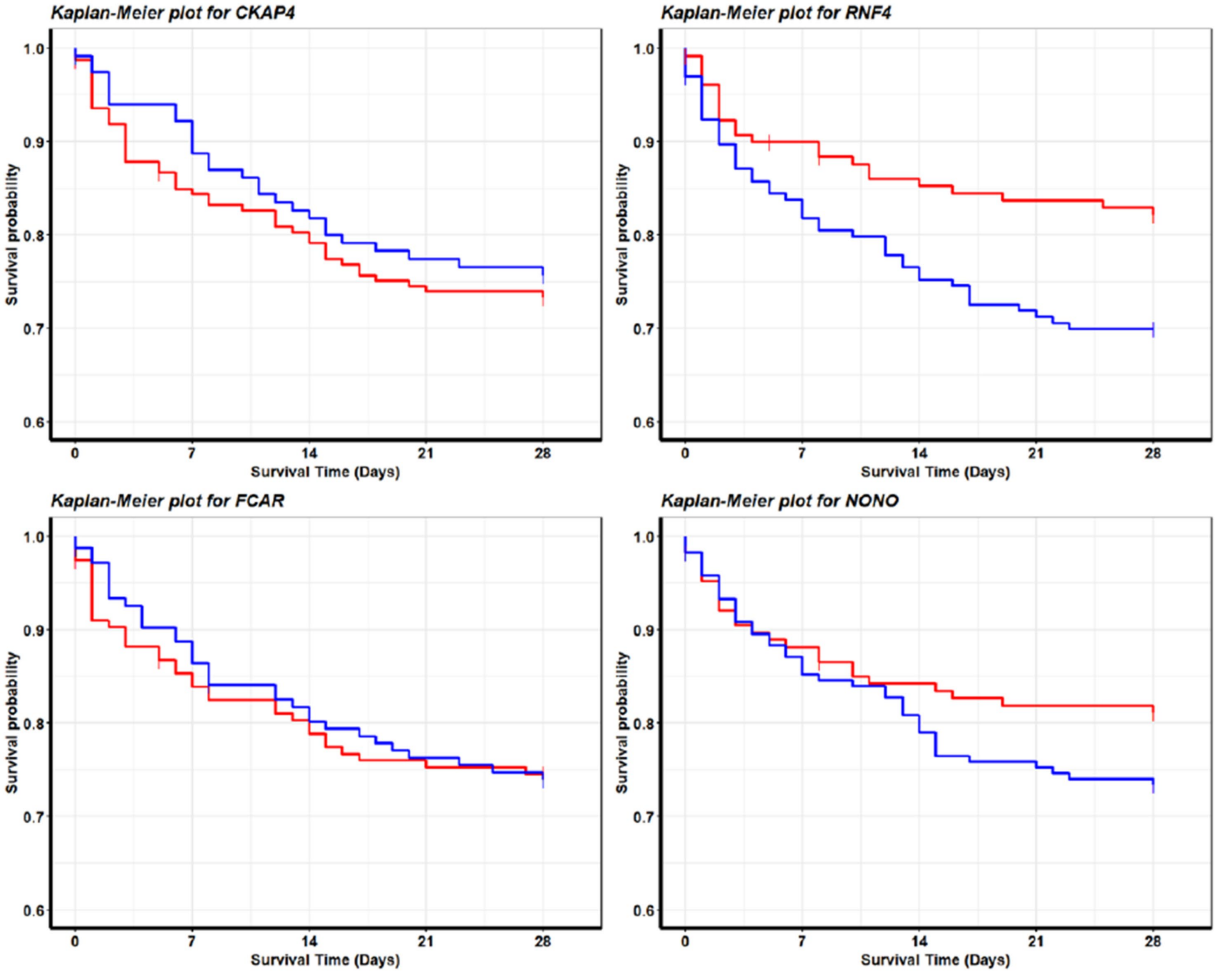
Survival analysis for sepsis patients. These Kaplan–Meier plots are produced with survival data from GSE65682. The red curve and blue curve represent patients with high expression and low expression, respectively. We define high as greater than the 70th quantile, low as less than 30th quantile, in the final analysis we included 456 out of 760 patients in the cohort. The patterns are consistent with the fitted model coefficient signs for GSE65682 in Table 4. We see that the higher the NONO (RNF4) expression values are, the longer the survival time will be; the lower the CKAP4 expression values will be, the longer the survival time will be. Interestingly, FCAR does not lead to a significant pattern, which is also consistent with the model fitting as in Table 4 (GSE65682) CF_2_ does not contain FCAR in its gene–gene interactions. These observations may lead to clues about sepsis genetic treatments and drug developments.

We note that for the seven datasets (GSE65682, GSE28750, GSE57065, GSE95233, GSE69528, GSE131761, and GSE154918) that recorded RNA expression collected in whole blood samples, their classifiers shared the same panel of 4 genes (CKAP4, RNF4, FCAR, and NONO) with the same core of three genes: CKAP4, RNF4, and FCAR. We also note that the classifiers of all but one of 1,413 these samples shared the same + or − signs for the core of three genes; the 1 sample that stood out was unique in its gene expression patterns (alternatively might be recording error), and a particular classifier was built to accommodate the difference. We argue that the consistency shown in the classifiers of different cohorts reflects an underlying pattern in the pathology of sepsis on the genomic level. In the case of these samples, a pattern consisting of upregulation of CKAP4 and FCAR along with downregulation of RNF4 decisively discriminates between sepsis patients and healthy samples. Such phenomena are also reflected in Figure 4 (survival probabilities).

#### The plasma samples of adult patients

Furthermore, we note that in 2 datasets (GSE49757 and self-collected) that recorded RNA expression of sepsis plasma samples, an opposite/reversed pattern could be observed for the same core of three genes. In these samples, the downregulation of CKAP4 and FCAR and the upregulation of RNF4 increase the risk of a sample being classified as “sepsis.”

#### The whole blood samples of pediatric patients

Finally, in the two pediatric datasets (GSE9692 and GSE13904), where RNA expression data was recorded from whole blood samples, the same expression pattern was observed with adult counterparts in the core of three genes. Also, a uniform pattern of downregulation of gene OGFOD3 increases the risk of sepsis.

#### Homogeneities across whole blood, plasma, adult patients, and pediatric patients

Overall, we report that a change in CKAP4, FCAR, and RNF4 expression patterns is a key feature of sepsis on the genomic level. In whole blood expression patterns, a consistent upregulation of CKAP4 and FCAR with downregulation of RNF4 showed a remarkably strong association with sepsis, reaching 100% accuracy, 100% sensitivity, and 100% specificity across different cohorts from a variety of socioeconomic backgrounds and ethnicity groups. The same pattern with the three genes (CKAP4, FCAR, and RNF4) could be extended to pediatric cohorts, while an opposite pattern was observed in adult plasma samples.

#### Heterogeneities between whole blood and plasma

We note that RNA expressions recorded from whole blood samples and plasma samples are expected to differ. They need to be balanced among cell-free RNAs and cell-led RNAs to remain healthy. We suspect that the opposite/reversed pattern may be attributed to the location where these genes are differentially expressed in a sepsis patient. We note that previous literature reported that CKAP4, RNF4, and FCAR had an association with immunity response. We hypothesize that the variations in gene expression in sepsis patients occur in specific cells in the blood and then reflect a pattern of upregulation of genes CKAP4 and FCAR in whole blood samples along with downregulation of RNF4. We hypothesize that this pattern coincides with an overwhelmed immune system. On the contrary, these cells are removed in plasma, and we observe different gene–gene interactions compared to those in the whole blood, with some coefficient signs changing in opposite directions. In addition, two other genes, PLEKHO1 and BMP6, are identified to interact with the miniature three genes. Such a phenomenon reveals that these three genes are pivotal in the progression of sepsis. They need to be balanced among cell-free RNAs and cell-led RNAs to remain sepsis-free. This finding is new in the literature and can potentially lead to new sepsis therapies.

#### Heterogeneities between adult and pediatric

Looking at GSE9692 in Table 4, we can immediately see that pediatric sepsis patients have simpler gene–gene interactions in whole blood compared to those of adult sepsis patients. The pediatric patients’ gene–gene interactions are from a pair of genes, e.g., CKAP4 and OGFOD3, RNF4 and OGFOD3, or CKAP4 and RNF4, to achieve 100% accuracy, while the adult patients need three genes. This observation may coincide with pediatric patients being more vulnerable to infection. In GSE13904, another gene, RNASE2, shows its pivotal function in sepsis infection, although it was not critical in adult patients.

### From sepsis to severe sepsis

We observe a clear relationship between gene expression and sepsis severity based on the plasma analysis from GSE49757 and the coefficient signs for the genes NONO, CKAP4, PLEKHO1, and BMP6 (as shown in Table 4). Specifically, increased expression of NONO and BMP6 correlates with a greater likelihood of severe sepsis. Conversely, lower expression of CKAP4 and PLEKHO1 also associates with increased sepsis severity, suggesting a complex, possibly non-linear interaction in their regulatory role. Among all sepsis patients, the expression patterns of CKAP4 and PLEKHO1 do not show significant linearity, further supporting the idea that certain genes contribute uniquely to disease progression depending on their interaction networks and expression thresholds. These results underscore the importance of interpreting gene expression profiles contextually, as the same gene may exhibit varied impacts across different stages of sepsis, necessitating a deeper exploration into the roles of NONO and BMP6 in immune response and cellular stress mechanisms.

### From sepsis to septic shock

The transition from sepsis to septic shock, as analyzed using GSE154918, highlights critical changes in the expression of the genes NONO, CKAP4, RNF4, and FCAR (refer to Table 4 for coefficients). Here, we observe that elevated expression of NONO, CKAP4, and FCAR strongly correlates with an increased risk of progression to septic shock. In contrast, RNF4 behaves inversely, where decreased expression is linked to heightened severity, suggesting a possible protective or compensatory role when downregulated. This gene signature, showing similarities to the patterns seen from healthy control to sepsis, suggests shared molecular pathways between the early onset and the severe stages of the condition. Furthermore, this alignment emphasizes the potential for certain genes, like NONO and CKAP4, to serve as robust markers across the full spectrum of sepsis severity.

Interestingly, while the plasma sample analysis provides valuable insights, the whole blood analysis offers even greater biological relevance. Whole blood contains a more comprehensive representation of the immune response and inflammatory signaling, making it more informative than plasma alone. This enhanced depth of information may provide additional layers of understanding of the pathophysiological shifts occurring from sepsis to septic shock, enabling more precise biomarker identification and therapeutic targeting.

### Peripheral blood mononuclear cells insights

In this section, we extend our analysis to a gene expression profiling dataset of peripheral blood mononuclear cells (PBMCs), i.e., GSE9960 (27). This dataset includes 54 sepsis patients and 16 healthy controls. Among 54 sepsis patients, the distributions are: 9 sepsis from non-infectious causes of systemic inflammatory response syndrome, 11 Gram-positive, 18 Gram-negative sepsis, and 10 mixed infections.

Using genes reported in Table 4, we can achieve ≥80% accuracy, sensitivity, and specificity. When including the core gene RNF4 in Table 4 and four additional genes (HTR2C, AC126474.2, CHCHD4, 244479_at), we can achieve 95.71% accuracy, 96.30% sensitivity, and 93.75% specificity using the following two max-logistic classifiers and their combination.

$\begin{array}{l} \mathrm{CF1}:-40.9212+8.0182^{*}\mathrm{LN}\;(\mathrm{RNF4}+1)\\ +52.3501^{*}\mathrm{LN}\;(\mathrm{HTR}2C+1)-36.7917^{*}\mathrm{LN}\;(\mathrm{CHCHD4}+1) \end{array}$

$\begin{array}{l} \mathrm{CF2}:-25.4168-0.8774^{*}\mathrm{LN}\;(\mathrm{RNF4}+1)-29.9838^{*}\mathrm{LN}\;\\ \left(\mathrm{AC}126474.2+1)+56.5659^{*}\mathrm{LN}\;(244479\_\mathrm{at}+1\right) \end{array}$

Here, HTR2C (5-Hydroxytryptamine Receptor 2C) is a Protein Coding gene. CHCHD4 (Coiled-Coil-Helix-Coiled-Coil-Helix Domain Containing 4) is a Protein Coding gene. AC126474.2 is an lncRNA gene. The probe set ID 244479_at has not yet been associated with a gene symbol.

Our analysis shows that the gene expression values obtained from whole blood, plasma, and PBMC samples exhibit significant differences, primarily influenced by sample composition, cell type proportions, and RNA degradation. As a result, selecting the appropriate sampling method is crucial. PBMC is more suitable for immunological studies, plasma is ideal for cell-free RNA research, and whole blood provides a more comprehensive but noisier expression profile.

## Discussion

Sepsis continues to be a major global health burden, with millions of cases each year and high mortality rates, particularly in intensive care units (ICUs) (1, 2). Although significant strides have been made in understanding the pathophysiology of sepsis, the long-term survival and quality of life of sepsis survivors remain critical challenges (3, 28, 29). Recent advances in transcriptomic profiling have provided a promising avenue for understanding the genomic underpinnings of sepsis and guiding the development of more targeted therapies (30). However, several inherent limitations in current transcriptomic studies of sepsis—such as small sample sizes, gene–gene interaction complexities, and inadequate differentiation between disease stages—limit their clinical utility. Numerous studies have targeted these limitations. Still, critical genetic biomarkers for sepsis remain unidentified, largely due to flawed animal models and patient selection over the past 30 years (17). Our study seeks to address this gap. In the meantime, subgroups of sepsis can significantly impact analysis results (31). Unlike traditional classification approaches, the max-logistic classifier inherently accounts for subgroups by employing competing classifiers. This method has been mathematically proven to be robust against study population heterogeneity in previous research on lung and colorectal cancers (22, 23).

In response to these challenges, we developed an AI-driven model that bridges classical methods and advanced machine learning to identify key differentially expressed genes (DEGs) involved in sepsis progression. Our model addresses the limitations of existing AI approaches, including the “black box” nature of many machine learning algorithms and the biases inherent in training processes (18, 20, 32). Unlike most AI models, which primarily emphasize inductive reasoning, our approach balances deductive, inductive, and abductive reasoning, allowing for more transparent and biologically interpretable results. This hybrid reasoning approach has been detailed in our previous work (21, 33), and the model has been validated in studies of various cancers and infectious diseases (22–25).

In our study, gene–gene interaction is not defined in the conventional biological sense, such as physical interaction, pathway co-membership, or co-expression. Instead, we introduce a mathematically grounded definition rooted in the max-logistic competing factor model. In this framework, gene–gene interactions are interpreted through the lens of competing combinations of genes, where specific subsets work together within a mathematical structure to compete for predictive power in distinguishing sepsis states.

These interactions are characterized by their coefficients (signs and magnitudes), combinatorial grouping, and their ability to outcompete other gene combinations in classification tasks. The presence of a gene in a dominant competing factor, along with the sign and strength of its coefficient, reflects its functional role in synergy or antagonism with other genes within that factor. This approach resembles quantum models in physics—where the outcome is determined by the configuration and interaction of components within a complex system—not simply by pairwise association.

While this perspective may differ from classical biological interaction models, it provides a reproducible, interpretable, and high-accuracy framework for capturing functional relationships among genes in disease progression, especially across heterogeneous populations. We hope this mathematical view complements and inspires further biological investigation into the mechanisms underlying these interactions.

### Key findings and control group considerations

One of the most important aspects of our study is the rigorous selection of control groups. Previous sepsis transcriptomic studies have predominantly compared sepsis patients to healthy controls, often with high diagnostic accuracy for specific gene sets. However, distinguishing healthy individuals from critically ill patients admitted to the ICU is relatively straightforward from a clinical perspective. The real challenge lies in identifying subpopulations within sepsis patients—such as those with severe sepsis or septic shock—that may respond differently to targeted treatments. By including these more clinically relevant subsets in our analysis, we aimed to address this gap in the literature.

Our model identified a set of eight critical DEGs, divided into three panels, which exhibit consistent patterns across different stages of sepsis. These patterns are particularly informative when comparing sepsis to more advanced conditions such as severe sepsis and septic shock. The inclusion of patients with severe sepsis and septic shock allowed us to more finely tune the gene expression profiles associated with worsening disease. For instance, the gene NONO showed varying expression trends depending on disease severity, with upregulation correlating with increased severity in certain populations, while downregulation had a similar effect in others. This underscores this gene’s complex, context-dependent role in sepsis pathology (Table 4).

### Analytic transparency and model development

Our AI model operates by systematically identifying linear combinations of genes that maximize the risk prediction of sepsis and its severe forms. Unlike traditional analyses, which often pool cohorts or fail to account for batch effects, we treated each of the 11 cohorts independently to avoid such issues. The diversity of the cohorts, which included over 1800 samples from different ethnic populations, age groups, and experimental platforms, presented a significant challenge. However, our max-logistic classifiers allowed for robust cohort-to-cohort cross-validation without the need for data pooling, thereby reducing the risk of biases due to batch effects (22).

Furthermore, our model development process was guided by a set of seven rules designed to ensure the identification of concise, precise, and generalizable DEGs. These rules exceed previous standards in the literature by emphasizing the importance of gene–gene interactions and the biological relevance of identified DEGs. By defining critical DEGs based on these stringent criteria, we have identified eight genes that demonstrate high sensitivity and specificity across diverse cohorts (34, 35). These findings provide a strong foundation for future applications of our model in precision medicine and risk stratification for sepsis patients.

### Biological relevance of identified DEGs

As discussed in the Introduction, the field of sepsis research has produced a vast array of published sepsis-related genes. This abundance of identified genes poses a significant challenge for precision medicine, as it complicates the development of targeted therapies. Our study addresses this issue by significantly reducing the gene set to a minimum single-digit level, which enhances the feasibility of precision therapeutic targeting. Importantly, we emphasize that the genes in our panel do not act in isolation; rather, they function interactively, demonstrating a synergistic relationship. This is a crucial advancement because while other researchers have observed the individual effects of some of these genes, our focus is on how their combined interactions drive sepsis pathophysiology. This gene–gene synergy underlines the novel approach of our research, offering a more comprehensive understanding of sepsis mechanisms and potential interventions. Below, we summarize the findings of the literature on the individual effects of three key genes in our set.

Each gene in our panels plays a critical role in sepsis pathophysiology. For example, FCAR encodes the Fc alpha receptor, which mediates immune responses by binding to immunoglobulin A (IgA) and promoting the release of pro-inflammatory cytokines (36–39). This receptor is essential in the immune defense against bacterial infections, particularly in the early stages of sepsis, where the immune system oscillates between hyperactivation and immunosuppression (40–43). The upregulation of FCAR in certain cohorts strongly distinguished sepsis patients from healthy controls, highlighting its potential as both a diagnostic biomarker and a therapeutic target (44, 45).

Similarly, RNF4, a RING finger E3 ubiquitin ligase, plays a pivotal role in the ubiquitination and proteasomal degradation of polysumoylated proteins. This process is critical in regulating inflammation, metabolism, and cell death—key mechanisms involved in sepsis pathogenesis (46–49). The downregulation of RNF4 in patients with septic shock suggests a potential protective role, where reduced degradation of substrates like PARP1 might mitigate excessive inflammatory responses, a hypothesis that warrants further investigation.

CKAP4, another gene identified in our panels, is a type II transmembrane protein that has been implicated in various inflammatory diseases. Recent studies have also highlighted its role as a receptor for the SARS-CoV-2 spike protein, linking it to both viral pathogenesis and thrombosis (50–53). Given its interaction with NF-κB, a key regulator of inflammation, the upregulation of CKAP4 in sepsis patients could provide a novel link between inflammatory signaling pathways and the progression of sepsis.

Although the individual roles of these three genes have been acknowledged in the progression of sepsis, it is critical to stress that none of them should be examined in isolation. The defining feature of our work is the demonstration of synergistic effects among the genes in our set, distinguishing our findings from previous studies, which have largely concentrated on individual gene fold changes. This singular focus on individual changes can be misleading, as it fails to capture the dynamic interactions that occur at the gene network level. The strength of our work lies in its exploration of these interactions, providing a more accurate and holistic view of sepsis biology. Future research should explore whether the downstream protein levels and pathways regulated by these genes contribute to sepsis severity, opening new avenues for potential therapeutic strategies.

### Ethnic variations and limitations

Our study also uncovered significant ethnic variations in gene expression patterns, further emphasizing sepsis pathophysiology’s complexity. For example, we observed that the downregulation of the gene NONO was linked to an increased risk of sepsis in Thai and Chinese cohorts, whereas in Australian and German cohorts, the opposite was true—upregulation of NONO was associated with a heightened sepsis risk. These findings strongly suggest that genetic or environmental factors may shape the way sepsis manifests in different populations, and crucially, these variations are not isolated to individual genes but are evident in the synergistic gene–gene interactions we have highlighted. The population-specific signatures of gene synergy warrant further investigation to deepen our understanding of the molecular mechanisms driving sepsis in diverse populations.

Additionally, our model provided indications of potential subtypes within the same cohort, potentially reflecting different infection sources or underlying biological mechanisms. While this is an intriguing finding, more data is necessary to fully confirm the subtypes and its implications for developing personalized treatment strategies. Identifying such subtypes could pave the way for more tailored approaches to sepsis care, accounting for both genetic background and the nature of the infection, thus enhancing the precision of medical interventions.

Despite the strengths of our model, several limitations must be acknowledged. The classification of sepsis has evolved over the past two decades, resulting in some inconsistencies in cohort categorization. Additionally, due to data availability, our study sourced data from the US, Europe, and China, which may limit the generalizability of our findings to much broader populations. Future studies should aim to include more diverse cohorts and explore the causality of gene expression changes in sepsis through longitudinal genomic analyses.

## Conclusion

Our study provides a novel and stringent approach to identifying critical DEGs in sepsis, utilizing an AI-driven model that outperforms existing methods in terms of accuracy, sensitivity, and specificity. While further validation is necessary, particularly in the form of wet lab studies, our findings offer new insights into the molecular mechanisms underlying sepsis and lay the groundwork for the development of precision diagnostic tools and targeted therapies. Sepsis remains one of the most pressing challenges in global health, and our work represents an important step toward overcoming this formidable disease.

## Data Availability

The datasets presented in this study can be found in online repositories. The names of the repository/repositories and accession number(s) can be found in the article/Supplementary material.
