# Supplementary material for: AI-driven discovery of minimal sepsis biomarkers for disease detection and progression: precision medicine across diverse populations
Source: Front Med (Lausanne). 2025 Jul 1;12:1521827. doi: 10.3389/fmed.2025.1521827 (PMC12259559; doi:10.3389/fmed.2025.1521827)
Supplement: Supplementary file 1 [file Presentation_1.pdf]

AI-Driven Discovery of Minimal Sepsis Biomarkers for Disease Detection and Progression: Precision Medicine Across Diverse Populations

(Supplementary)

Qiyuan Su[1], Jingtao Huang, PhD[2], Yunlong Zhang[3], Zhou Liu[3], Zhihua Lv, MD[2], Chunming Zhang, PhD[4], Chengxiu Ling, PhD[1], Hanwen Su, MD[2], Liying Zhan, MD[3], Zhengjun Zhang, PhD[5,6,7]

[1] Wisdom Lake Academy of Pharmacy, Xi'an Jiaotong-Liverpool University, Suzhou, Jiangsu, China; [2] Department of Clinical Laboratory, Institute of Translational Medicine, Renmin Hospital of Wuhan University, Wuhan, China; [3] Department of Critical Care Medicine, Renmin Hospital of Wuhan University, Wuhan, China; [4] Department of Statistics, University of Wisconsin, Madison, WI, USA; [5] School of Economics and Management, and MOE Social Science Laboratory of Digital Economic Forecasts and Policy Simulation, University of Chinese Academy of Sciences; [6]Center for Forecasting Sciences, Chinese Academy of Sciences, Beijing, China; [7] Department of Statistics, University of Wisconsin, Madison, WI, USA

This supplementary file provides details of public data descriptions, analytical procedures, mathematical formulas, definitions and criteria of critical genes.

Public Data acquisition

This study used ten public datasets, all obtained from the National Center for Biotechnological Information's (NCBI) Gene Expression Omnibus (GEO) database. Keywords "sepsis", "septic shock", and "homo sapiens" were utilized when searching for datasets. Two datasets (GSE9692, GSE13904) were pediatric cohorts from the US, while others were adult cohorts from the US, Australia, France, Spain, and Germany. The validation dataset was self-collected in a Chinese cohort. An overview of information on the datasets is presented in Table 1.

The first seven datasets consist of publicly available whole blood samples from adult patients. The first dataset, sourced from NCBI's GSE65682, includes samples from a North American adult cohort, with 761 severe pneumonia/sepsis cases and 41 healthy controls. In this study, blood was

collected from intensive-care units (ICUs), and total RNA was extracted using the PaxGene Blood RNA kit (Qiagen, Netherlands). Gene expression was measured using the Affymetrix Human Genome U219 Array platform.[1] The DEGs are RMA normalized log2 transformed values, as processed by the original GEO dataset contributors.

The second dataset, NCBI's GSE28750, is from an Australian adult cohort, containing 21 sepsis samples (classified using the Sepsis 2.0 International Consensus Definition from 2001) and 20 healthy controls. RNA was extracted from whole blood with the PaxGene Blood RNA kit (PreAnalytix, Switzerland), and gene expression was quantified on the Affymetrix Human Genome U133 Plus 2.0 Array platform.[2] The DEGs are RMA values, as processed by the original GEO dataset contributors.

The third dataset, NCBI's GSE57065, represents a European adult cohort with 28 ICU patients at the onset of septic shock. Blood was sampled at three time points: within 30 minutes, 24 hours, and 48 hours after the shock, with samples compared to 25 healthy volunteers. RNA extraction was performed using PaxGene Blood RNA kits (PreAnalytix, Germany), and gene expression was analyzed with the Affymetrix Human Genome U133 Plus 2.0 Array platform.[3] The DEGs are RMA signal intensity (log base 2) , as processed by the original GEO dataset contributors.

Similarly, the fourth dataset, NCBI's GSE95233, from a European adult cohort, includes 51 septic shock patients and 22 healthy controls. Patients were sampled twice: upon admission and again on Day 2 or Day 3. This dataset comes from the same study as GSE57065 but focused on a different aspect. The gene expression methods and platform used were identical.[3] The DEGs are RMA signal intensity (log base 2) , as processed by the original GEO dataset contributors.

The fifth dataset, NCBI's GSE69528, from an American adult cohort, contains 83 sepsis and 55 control samples. RNA was isolated with the Tempus Spin Isolation kit (Applied Biosystems), and gene expression was assessed on the Illumina HumanHT-12 V4.0 expression beadchip platform.[4] The DEGs are Quantile normalized, as processed by the original GEO dataset contributors.

The sixth dataset, NCBI's GSE131761, is from a Spanish adult cohort comprising 114 sepsis and 15 control samples. In this study, RNA was extracted using the PAXgene Blood RNA System (PreAnalytix, Switzerland) and the RNeasy Mini Kit (Qiagen, Germany). Gene expression was measured using the Agilent-026652 Whole Human Genome Microarray 4x44K v2 platform.[5] The DEGs are normalized signal intensity, as processed by the original GEO dataset contributors.

The seventh dataset, NCBI's GSE154918, contains samples from an Australian and German adult cohort, with 79 sepsis and 26 control samples. RNA extraction was performed using the PAXgene Blood RNA Kit, and gene expression was quantified on the Illumina HiSeq 4000 platform.[6] The DEGs are log2 normalized values, as processed by the original GEO dataset contributors.

The next two datasets come from pediatric patients' whole blood samples. The eighth dataset, NCBI's GSE13904, involves a North American pediatric cohort with 209 samples classified under systemic inflammatory response syndrome (SIRS), sepsis, and septic shock (following the Sepsis 1.0 International Consensus Definition from 1992), alongside 18 healthy controls. RNA was isolated using the PaxGene Blood RNA kit (PreAnalytiX, USA), and gene expression was analyzed on the Affymetrix Human Genome U133 Plus 2.0 Array platform.[7] The DEGs are normalized to mean of controls, as processed by the original GEO dataset contributors.

The ninth dataset, NCBI's GSE9692, also from a North American pediatric cohort, includes 30 sepsis and 14 control samples. RNA was extracted using the PaxGene Blood RNA System (PreAnalytiX; Qiagen/Becton Dickson, USA), and gene expression was analyzed with the Human Genome U133 Plus 2.0 GeneChip platform.[8] The DEGs are normalized to mean of controls, as processed by the original GEO dataset contributors.

The final two datasets are derived from plasma samples taken from adult patients. The tenth dataset, NCBI's GSE49757, from a North American adult cohort, includes 37 sepsis and 19 control samples. It is a subset of a larger study found in GSE49758. In this study, RNA was extracted using the RNeasy Mini kit (Qiagen, Netherlands), and gene expression was assessed on the Illumina HumanHT-12 V4.0 expression beadchip platform.[9] The DEGs were normalized using quantile normalization, as processed by the original GEO dataset contributors.

Of the ten public datasets, seven derived from adult whole blood samples allow for cohort-to-cohort cross-validation. The two pediatric datasets are also naturally suited for cross-validation. To validate findings from the only public plasma dataset, we collected a new cohort from ICU patients at Wuhan University Renmin Hospital, China, which included 32 sepsis and 18 healthy control samples. In this cohort, RNA was extracted using the HYCEZMBIO Serum/Plasma RNA Kit (HuiYuCheng Biotechnology, China), and gene expression was quantified using real-time quantitative polymerase chain reaction (rt-QPCR) on the Roche Light Cycler 480 platform. Details of the procedures for collecting this dataset are provided in the supplementary materials. The DEGs' gene expression levels were normalized to the housekeeping gene β-actin as an internal control, and relative expression was calculated.

Analytical method

The AI analytical model used in this study specializes in nonlinear prediction and classification, and its detailed description and theoretical rationale have been published in previous literature. This model aims to search for the most concise subset of critical differentially expressed genes (DEGs) with the highest classification power. The criteria we used for the definition of critical DEGs were based on the existing literature.[10,11] However, we modified the recommendations to fit our research context and developed the following specified rules:

1) The critical DEG subset should contain no more than 15 genes (such a number has been widely reported in the literature).
2) The critical DEG subset should exhibit an overall accuracy of at least 95% in at least three distinct study cohorts, with a total of at least 1,000 patients/subjects.
3) The critical DEG subset should exhibit an overall accuracy of 100% in at least one study cohort, with a minimum of 10 subjects.
4) At least one gene shows the same sign (+ / −) and serves the same function in each study cohort.
5) The critical DEG subset should show at least 80% accuracy in any given cohort, with either sensitivity or specificity values exceeding 75%.
6) The competing classifiers should include the smallest number of genes possible so that rules 1) through 5) are not violated.
7) The number of competing classifiers should be minimized to avoid redundancy.

We remark that many published works didn't involve cohort-to-cohort validations, i.e., they cannot satisfy 2), 3), and 5). Many existing machine-learning approaches lack interpretability and don't satisfy 4). Most published work couldn't apply to heterogeneous populations, e.g., the eleven cohorts in this paper, so their evidence might be biased. Rules 6) and 7) ensure the model's conciseness and help avoid overfitting.

To further scrutinize the results, we added a heterogeneous extension to the max-logistic regression model to support better consistency between analyses of different cohorts. The theoretical rationale of competing risk classifiers for heterogeneous populations is described below.

Suppose there are K cohorts with their binary (1 for sepsis, 0 for healthy) response variables being $Y_{(1)} \dots , Y_{(K)}$. In the kth cohort, we have n_k observed response, denoted as

$$Y_{(k)} = \left(Y_{1k}, Y_{2k}, \dots, Y_{n_k k}\right)^{\top}. \quad \text{(s1)}$$

Each of the Y_{ik} may be related to G groups of genes by

$$\Phi_{ijk} = \left(X_{i,j_1,k}, X_{i,j_2,k}, \dots, X_{i,j_{g_j},k}\right), \quad where\, j = 1,2, \dots, G,\ and\, g_j \geq 0, \quad \text{(s2)}$$

where g_j stands for the number of genes grouped into the jth group.

The competing (risk) factor classifier for the kth cohort (outcome variable) is defined as

$$\log \frac{p_{ik}}{1-p_{ik}} = \max(\beta_{01k} + \Phi_{i1k}\beta_{1k}, \beta_{02k} + \Phi_{i2k}\beta_{2k}, \dots, \beta_{0Gk} + \Phi_{iGk}\beta_{Gk}), \quad \text{(s3)}$$

where β_{0jk}'s are intercepts, β_{jk} is a g_j-dimensional coefficient vector associated with the jth grouped genes. We see that β_{jk} characterizes the contribution of each predictor in the jth group to the outcome/risk, and $\beta_{0jk} + \Phi_{ijk}\beta_{jk}$ is called the jth competing risk factor, i.e., j^{th} signature.

Remark 1: With $\beta_{0jk} = -\infty, j = 2, \dots, G$, Eq. (s3) is reduced to the classical logistic regression classifier. Intuitively, every component of $\beta_{0jk} + \Phi_{ijk}\beta_{jk}, j = 1, \dots, G$ is a risk factor for a patient to be sepsis, and the highest risk is from the largest one, i.e., these risk factors compete against each other to win out to make the final effect, i.e., to determine whether a patient is sepsis. As such, they are called competing risk factors.

The unknown parameters are estimated from $\left(\beta_{(k)} = (\beta_{01k}, \beta_{1k}, \dots, \beta_{0Gk}, \beta_{Gk})\right)$

$$\left(\hat{\beta}_{(k)}, \hat{S}\right) = argmin_{\beta_{(k)}, S_j \subset S, j=1,2,\dots G} \sum_{i=1}^{n_k} [I(p_{ik} \leq 0.5) I(Y_{ik} = 1) + I(p_{ik} > 0.5) I(Y_{ik} = 0)], \quad \text{(s4)}$$

where 0.5 is a probability threshold value that is commonly used in binary machine learning classifiers, $I(\cdot)$ is an indicator function, p_{ik} is defined in Eq. (s3), $S = \{1, 2, \dots, n_G\}$ is the index set

of all genes, $S_1 = \{1_1, 1_2, \dots, 1_{g1}\}$, $S_2 = \{2_1, \dots, 2_{g2}\}$, … , $S_G = \{G_1, \dots, G_{gG}\}$ are index sets corresponding to Eq. (s2), and $\hat{S} = \{1_1, 1_2, \dots, 1_{g1}; 2_1, \dots, 2_{g2}; \dots; G_1, \dots, G_{gG}\}$ is the final gene set selected in the final classifiers.

To introduce sparsity for both the number of variables (genes) and the number of groups (competing factors, signatures) into the model, the following optimization problem with penalties is considered.

$$(\hat{\beta}, \hat{S}, \hat{G}) = argmin_{\beta_{(k)}, S_j \subset S, j=1,2,\dots G} \left\{ (1 + \lambda_1 + |S_u|)^{\sum_{k=1}^{K} \sum_{i=1}^{n_k} [I(p_{ik} \le 0.5) I(Y_{ik}=1) + I(p_{ik} > 0.5) I(Y_{ik}=0)]} + \lambda_2 \left(|S_u| - \frac{|S_u| + G - 1}{(|S_u| + 1) \times G - 1} \right) \right\} \quad \text{(s5)}$$

where S_u is the union set of $\{S_j\}_{j=1}^{G}$, $|\cdot|$ is the cardinality. Tuning parameters λ_1 and λ_2 are both nonnegative. Component $\frac{|S_u|+G-1}{(|S_u|+1)\times G-1}$ is monotone decreasing in both $|S_u|$ and G. Other properties of competing models are described in previous literature.[12-15]

Remark 2: The smallest subset and smallest number of signatures (S4) property of Eq. (s4) and its capability to simultaneously classify multiple heterogeneous populations with common variables (genes) make the new competing risk factor classifier different from existing ones.[14]

Remark 3: The details of computational steps were described in previous literature, and demo MATLAB codes are publicly available online. Eq. (s5) is an optimization problem with extremely high computational complexity, as it integrates integer programming, combinatorial optimization, and continuous optimization. Therefore, in practice and for this study, we adopted the Monte Carlo approach to solve Eq. (s5). Here, we restate the computational guidance for this optimization problem elaborated in our earlier work with slight modifications according to our setting and references.[16]

Set G = 1, $|S_1|$ = 3 (or 4, 5). Pre-define sensitivity level sen = 0.6 (or 0.7, 0.8, 0.9) and specificity level spe = 0.90 (or 0.95). The initial selection of pre-defined sen and spe levels may rely on previous literature or researchers' target.

Perform 50,000 (or larger numbers) random draws of sets of S_1 genes.

Evaluate each set of S_1 genes in Step 2 and calculate the sensitivity and specificity. These genes are recorded if both are larger than the pre-specified sen and spe levels. This step helps to filter essential genes and reduce the number of genes in scope.

If the number of recorded genes in Step 3 is greater than 30 (or 25, 20, depending on the target of gene number shrinking), raise sen and spe values and repeat Step 2 with random draws among recorded genes in Step 3. Repeat Step 3 to further filter the recorded genes.

Repeat Step 4 until the number of recorded genes is less than 30 (or 25, 20). These recorded genes are considered candidate genes.

Set G=3 (or 2, 4, 1), $|S_j|$ = 3 (2, 4, 1). Perform 50,000 (or larger numbers) random draws of sets of S_i genes among the candidate genes selected in Step 5.

Report the best results with the S4 properties.

Reference

1. Scicluna BP, Klein Klouwenberg PM, van Vught LA, et al. A molecular biomarker to diagnose community-acquired pneumonia on intensive care unit admission. *Am J Respir Crit Care Med*. Oct 1 2015;192(7):826-35. doi:10.1164/rccm.201502-0355OC
2. Sutherland A, Thomas M, Brandon RA, et al. Development and validation of a novel molecular biomarker diagnostic test for the early detection of sepsis. *Crit Care*. Jun 20 2011;15(3):R149. doi:10.1186/cc10274
3. Tabone O, Mommert M, Jourdan C, et al. Endogenous Retroviruses Transcriptional Modulation After Severe Infection, Trauma and Burn. *Front Immunol*. 2018;9:3091. doi:10.3389/fimmu.2018.03091
4. Pankla R, Buddhisa S, Berry M, et al. Genomic transcriptional profiling identifies a candidate blood biomarker signature for the diagnosis of septicemic melioidosis. *Genome Biol*. 2009;10(11):R127. doi:10.1186/gb-2009-10-11-r127
5. Martinez-Paz P, Aragon-Camino M, Gomez-Sanchez E, et al. Distinguishing septic shock from non-septic shock in postsurgical patients using gene expression. *J Infect*. Aug 2021;83(2):147-155. doi:10.1016/j.jinf.2021.05.039
6. Herwanto V, Tang B, Wang Y, et al. Blood transcriptome analysis of patients with uncomplicated bacterial infection and sepsis. *BMC Res Notes*. Feb 27 2021;14(1):76. doi:10.1186/s13104-021-05488-w
7. Wong HR, Cvijanovich N, Allen GL, et al. Genomic expression profiling across the pediatric systemic inflammatory response syndrome, sepsis, and septic shock spectrum. *Crit Care Med*. May 2009;37(5):1558-66. doi:10.1097/CCM.0b013e31819fcc08
8. Cvijanovich N, Shanley TP, Lin R, et al. Validating the genomic signature of pediatric septic shock. *Physiol Genomics*. Jun 12 2008;34(1):127-34. doi:10.1152/physiolgenomics.00025.2008
9. Khaenam P, Rinchai D, Altman MC, et al. A transcriptomic reporter assay employing neutrophils to measure immunogenic activity of septic patients’ plasma. *Journal of Translational Medicine*. 2014/03/11 2014;12(1):65. doi:10.1186/1479-5876-12-65
10. McDermaid A, Monier B, Zhao J, Liu B, Ma Q. Interpretation of differential gene expression results of RNA-seq data: review and integration. *Brief Bioinform*. Nov 27 2019;20(6):2044-2054. doi:10.1093/bib/bby067

11. Røsok Ø, Sioud M. Discovery of Differentially Expressed Genes. In: Sioud M, ed. *Target Discovery and Validation Reviews and Protocols: Volume 1, Emerging Strategies for Targets and Biomarker Discovery*. Humana Press; 2007:115-129.
12. Zhang ZJ. Functional Effects of Four or Fewer Critical Genes Linked To Lung Cancers and New Subtypes Detected by a New Machine Learning Classifier. *Journal of Clinical Trials*. 2021;
13. Zhang ZJ. Lift the Veil of Breast Cancers Using 4 or Fewer Critical Genes. *Cancer Inform*. Feb 2022;21doi:Artn 117693512210763601 0.1177/11769351221076360
14. Zhang ZJ. The Existence of at Least Three Genomic Signature Patterns and at Least Seven Subtypes of COVID-19 and the End of the Disease. *Vaccines-Basel*. May 2022;10(5)doi:ARTN 761
10.3390/vaccines10050761
15. Cui QR, Xu YQ, Zhang ZJ, Chan V. Max-linear regression models with regularization. *J Econometrics*. May 2021;222(1):579-600. doi:10.1016/j.jeconom.2020.07.017
16. Liu YJ, Xu YQ, Li XX, et al. Towards precision oncology discovery: four less known genes and their unknown interactions as highest-performed biomarkers for colorectal cancer. *Npj Precis Oncol*. Jan 20 2024;8(1)doi:ARTN 1310.1038/s41698-024-00512-1
